# Neuromodulation attenuates bladder hyperactivity in a rat cystitis model

**DOI:** 10.1186/1471-2490-13-70

**Published:** 2013-12-06

**Authors:** Xin Su, Angela Nickles, Dwight E Nelson

**Affiliations:** 1 Medtronic, Inc, Neuromodulation Research, 7000 Central Avenue, Minneapolis, MN 55432, USA; 2 Medtronic, Inc, Physiology Research Laboratory, 11520 Yellow Pine St, Coon Rapids, MN 55448, USA

**Keywords:** Electrical stimulation, Spinal nerve, Bladder, Micturition, Acetic acid, Neuromodulation

## Abstract

**Background:**

We investigated the regulation of urinary bladder function by electrical stimulation of the L6 spinal nerve (SN) using cystometry in normal rats and in rats with cystitis induced by intravesical infusion of dilute acetic acid.

**Methods:**

In anesthetized rats, a cannula was placed into the bladder dome for saline/acetic acid infusion and intravesical pressure monitoring. Threshold pressure (TP), basal pressure (BP) and inter-contraction interval (ICI) were measured from the bladder pressure recording and void volume (VV) was measured by weighing the voided fluid.

**Results:**

Comparison of cystometrograms obtained with infusion of saline or acetic acid showed that acetic acid decreases TP, ICI and VV. These excitatory effects, characteristic of acetic acid induced bladder hyperactivity, were significantly reversed by bilateral SN stimulation (P <0.05, vs pre-stimulation, Student t-test). In saline perfused rats, one hour of bilateral SN stimulation at 10 Hz and at motor threshold (0.19 ± 0.01 milliamps) increased ICI (265%) and VV (217%). In rats perfused with acetic acid, the corresponding increases produced by SN stimulation were 350% for ICI and 383% for VV. The percentage increases in the acetic acid-treated group were not significantly higher than those in saline-treated group.

**Conclusions:**

Using continuous flow cystyometry, we find that SN stimulation can produce effects on micturition consistent with its effects on isovolumetric model, and consistent with the therapeutic effect observed with InterStim® therapy in overactive bladder patients. Although the effect of SN stimulation was slightly greater in bladder irritated over normal rats, the difference was not statistically significant.

## Background

Electrical stimulation of the sacral spinal nerve (SN, S3), has been established as an effective treatment for patients with urge incontinence, increased urinary frequency and retention. Objective measures of therapeutic efficacy can be obtained. For example, chronic sacral root neuromodulation for refractory urge incontinence results in a 15% increase in cystometric capacity [1]. However, it is not feasible to perform these studies routinely. Clinical cystometry is time consuming, and large numbers of patients must be studied because of the variability of the results. Therefore efficacy criteria are usually based on improvement of subjective symptoms, such as urgency; treatment is considered successful if symptoms improve by at least 50%.

Evaluation of the efficacy of sacral neuromodulation in animal models is different. Subjective symptoms such as urgency are impossible to measure in animals. On the other hand, objective cystometric parameters are generally more reliable since experimental conditions can be controlled, and subject variability is lower. SN stimulation decreased voiding frequency from 10.9 to 6.2 per hr (~76% increase in bladder capacity) in rats with cyclophosphamide-induced cystitis [2].

Utilizing an in vivo rat model of bladder rhythmic contraction (BRC) the optimal stimulation parameters (current intensity and frequency) have been identified for SN stimulation induced inhibition of bladder contractions under isovolumetric conditions. Bilateral stimulation of the L6 SN attenuates bladder contraction frequency. Maximal inhibition was observed at a stimulation frequency of 10 Hz; the preparation showed sensitivity to SN stimulation when stimulated at the motor threshold T_mot_, [3,4].

BRC has facilitated rapid screening of acute responses to stimulation in anesthetized animals. However, only bladder contraction, not actual voiding is measured. Using continuous open cystometry allows the assessment of the effects of SN stimulation on several important parameters of urodynamic function. It is necessary to confirm that the optimal parameters of SN stimulation identified in the BRC model, will also affect actual micturition, either in normal rats or in rats with detrusor overactivity. These cystometric experiments target more directly the mechanisms by which neuromodulation acts to relieve the symptoms of overactive bladder.

Stimulation with parameters that produced maximum inhibition of isovolumetric contractions in the cat produced a 19% increase in cystometric capacity [5]. The duration of stimulation was variable depending on the length of storage phase in each voiding cycle and the reported effect on bladder capacity was induced by combined responses to neuromodulation of SN, pudendal nerve and the dorsal nerve of the penis [5].

In this study we describe the cystometric effects of electrical stimulation of SN using acetic acid induced cystitis in the rat [6,7]. Based on our parameter optimization studies in the BRC model [3,4], SN stimulation was applied bilaterally at a frequency of 10 Hz using a clinically relevant current intensity equal to the motor threshold (T_mot_).

## Methods

Male Sprague–Dawley rats weighing 200–300 g were anesthetized with urethane (Sigma-Aldrich, St. Louis, MO; two i.p. injections, 4 min apart, total 1.2 g/kg). Anesthetized rats were maintained at 37°C with a heating pad (COAX-3 T, Viking Medical, Medford Lakes, NJ) during the studies and were euthanized by CO_2_ asphyxia upon completion of the experiment. The experimental protocols were approved by the Institutional Animal Care and Use Committee of Medtronic (Minneapolis, MN).

An abdominal incision was made to provide access to the urinary bladder. A catheter (PE-90) was inserted into the bladder through a small incision in the apex of the dome and secured using a purse string suture. The other end of the catheter was externalized and the skin incision was closed with suture. To deliver electrical stimulation, two wire electrodes were separately placed beneath each L6 SN [8]. The skin around the dorsal sacral and thoracic area was shaved and a dorsal midline incision was made from approximately L3 to S2, the L6/S1 posterior vertebral processes were exposed. The S1 processes were removed and the L6 nerve trunks localized caudal and medial to the sacroiliac junction. The wire electrode was a bared portion of teflon-coated, 40-gauge, stainless steel wire (Cooner Wire Co., Chatsworth, CA). After the electrode was positioned under each nerve, silicone adhesive (Kwik-Cast, World Precision Instruments, Inc, Fl, USA) was applied to cover the wire around the nerve, and the skin incision was closed with suture. The electrode was connected to a Grass S88 stimulator (Grass Medical Instruments) through a stimulus isolation unit (SIU-BI, Grass Medical Instruments). A needle electrode placed under the skin of the tail served as the electrical ground.

Biphasic pulses (pulse width 0.1 ms) at motor threshold intensity (T_mot_) were used to stimulate the SN at frequency of 10 Hz. Electrical stimulation of the SN evoked hind-toe twitches and/or pelvic floor muscle contractions. In each rat, T_mot_ was defined as the lowest intensity to evoke the first, barely discernable muscle contraction. T_mot_ from the right and left SN stimulation in an individual rat may not be identical, therefore the stimulus intensities on the two sides were adjusted independently.

Following surgery, rats were placed in restraints (Braintree Scientific) located above an electronic balance positioned to measure voided urine volume. Urinary bladder catheters were made using PE-90 tubing and connected to a pressure transducer and a saline infusion pump (Harvard Instruments) via a three-way connector. Bladder pressure was viewed and recorded via Chart software through a PowerLab data acquisition system (ML880/P, AD Instruments). Bladders were continuously infused with room temperature saline (control), or 0.3% acetic acid (irritated bladder) at a rate of 50 μLmin^-1^ for the duration of the studies. A 2-hr infusion was used as an equilibration period allowing bladder parameters to stabilize. After equilibration, a 2-hr of control/basal urodynamics was obtained prior to spinal nerve stimulation for 1 hr. Urodynamics were continually assessed for an additional 2 hrs following termination of nerve stimulation.

Urodynamic response to 0.3% acetic acid irritation (n = 12) was first compared with that to a 5-hr saline infusion (n = 8) to establish a stable timeline of bladder irritation. For neurostimulation, animals were divided into four groups: 1) control non-irritated bladder (saline infusion) without SN stimulation (n = 8), 2) control non-irritated bladder with SN stimulation (n = 11), 3) irritated bladder (acetic acid infusion) without SN stimulation (n = 9), and 4) irritated bladder with SN stimulation (n = 12).

Cystometry parameters were assessed including basal bladder pressure (BP, mmHg), maximum pressure (MP, mmHg), threshold pressure (TP, mmHg), void volume (VV, ml), residual volume (RV), and inter-contraction interval (ICI, min). ICI was measured as the time in min between micturition events and VV as the voided volume in mL per micturition event. TP, MP and BP were measured before each micturition occurred, the maximum pressure reached during voiding and after voiding in mmHg. Residual volumes were assessed mathematically as the difference between the volume infused (interval X 50 μL·min^-1^) and volume voided for a given cystometrogram.

### Data analyses

Data were calculated in 30-min bins, each having four control periods, two periods during stimulation, and four periods after stimulation. In addition, data to nerve stimulation were also normalized according to the mean during the last 30-min prior to stimulation to compare response sensitivities in saline or acetic acid perfused rats. Data were expressed as averages with associated standard errors, and statistics were performed by using Prism 5 (GraphPad Software, Inc., San Diego, CA). Student t-test (P < 0.05) was utilized to compare means in the same treatment group or among the different groups. Time course for the response to acetic acid irritation was analyzed using repeated measures ANOVA (Prism 5, GraphPad Software, Inc., San Diego, CA). Bonferroni post-hoc test was used to determine the statistical significance between different time points.

## Results

### Urodynamic response to 0.3% acetic acid irritation

**Figure 1:**
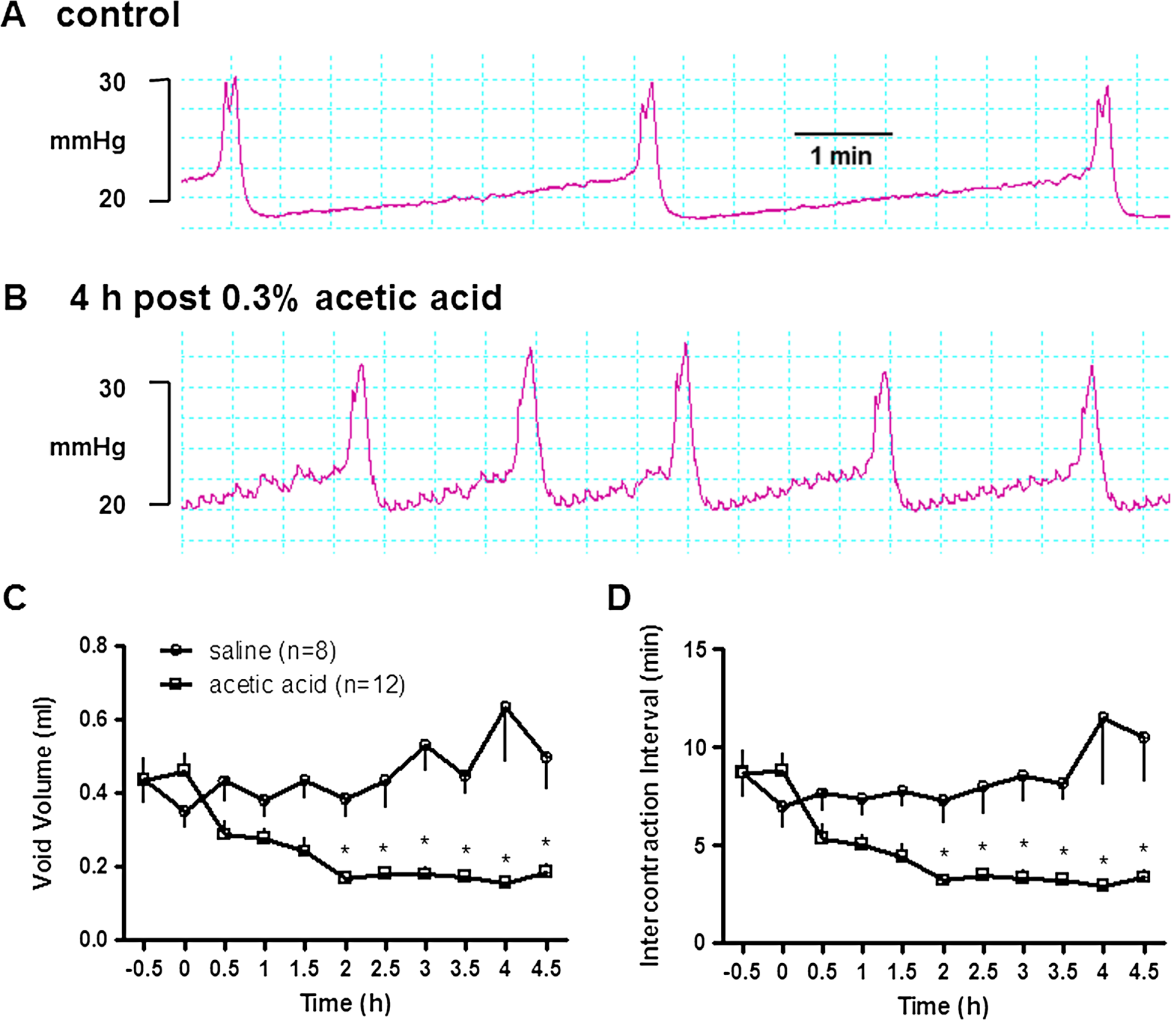
**The urodynamic response to intravesical administration of 0.3% acetic acid. A and B**. Bladder pressure recordings (mmHg) before and during intravesical injection of 0.3% acetic acid. **C and D**. Time course of the response on rat urodynamics to intravesical administration of 0.3% acetic acid. *P < 0.05, Repeated measures ANOVA, Bonferroni post test.

In continuous-filling cystometry, measurement of urodynamic parameters during a 5-hour saline infusion showed no significant time-related change in any of the parameters. Intra-vesical infusion of 0.3% acetic acid had an excitatory effect on micturition. A representative cystometrogram before and 4 hr after acetic acid infusion is shown in Figure 1 (A and B).

The excitatory effect was reflected by a time-dependent decrease in the VV (Figure 1C) and ICI (Figure 1D) after intravesical administration of acetic acid. Such excitatory effects stabilized at 2 hrs after initiation of acetic acid and lasted for at least 4 hrs. The values for VV, ICI, and TP during the infusion of acetic acid were significantly different than the values before infusion (Table 1, *P < 0.05, Student t-test). Repeated measures ANOVA analysis demonstrates acetic acid treatment was associated with a significant reduction in void volume and inter-contraction interval (P < 0.001, acetic acid vs saline). Acetic acid had no significant effect on basal pressure and peak maximum pressure.

**Table 1:** Cystometry parameters following intravesical infusion of saline or acetic acid

	**BP (mmHg)**	**MP (mmHg)**	**TP (mmHg)**	**VV (ml)**	**RV (ml)**	**ICI (mim)**
Control	11.32 ± 2.77	26.42 ± 3.53	16.17 ± 3.04	0.35 ± 0.04	0 ± 0.01	6.95 ± 0.91
2-hr saline	10.60 ± 2.44	25.98 ± 3.10	15.83 ± 2.90	0.39 ± 0.04	-0.02 ± 0.02	7.29 ± 1.01
4-hr saline	10.53 ± 2.25	27.44 ± 3.82	16.12 ± 3.42	0.57 ± 0.16	-0.06 ± 0.03	11.50 ± 3.16
Control	14.14 ± 1.52	27.66 ± 1.76	18.18 ± 1.93	0.46 ± 0.05	-0.02 ± 0.02	8.78 ± 0.88
2-hr acetic acid	14.64 ± 1.86	27.60 ± 1.76	15.91 ± 1.81*	0.17 ± 0.02*	-0.01 ± 0	3.19 ± 0.32*
4-hr acetic acid	14.87 ± 1.88	26.14 ± 2.20	17.10 ± 2.17	0.15 ± 0.02*	-0.01 ± 0.01	2.93 ± 0.33*

BP: basal pressure, MP: maximum pressure, TP: threshold pressure, VV: void volume, RV: residual volume, ICI: intercontraction interval. *P < 0.05, paired Student t-test for intragroup comparison to control “before acetic acid”.

### SN stimulation inhibits the excitatory response to intravesical administration of acetic acid

The threshold current (T_mot_) at which first visible motor contraction occurred was 0.19 ± 0.01 mA (n = 62; range: 0.01 – 0.40 mA; 95% confidential interval: 0.16 – 0.22 mA).

SN stimulation at T_mot_ significantly attenuated the acetic acid induced excitatory effect on bladder micturition reflex. Table 2 shows mean changes in the values for the cystometric parameters during 1 hr SN stimulation.

**Table 2:** Changes in cystometry parameters to spinal nerve stimulation for 1 hr

		**BP (mmHg)**	**MP (mmHg)**	**TP (mmHg)**	**VV (ml)**	**RV (ml)**	**ICI (mim)**
Saline	0.5 hr before	10.59 ± 2.47	23.06 ± 2.26	14.88 ± 2.57	0.43 ± 0.04	-0.05 ± 0.02	7.78 ± 0.70
	-stim	10.88 ± 2.41	25.47 ± 2.73	15.20 ± 2.99	0.41 ± 0.05	-0.03 ± 0.02	7.64 ± 1.01
	0.5 hr after	11.22 ± 2.52	26.21 ± 3.08	15.68 ± 3.41	0.53 ± 0.06	-0.10 ± 0.03	8.53 ± 1.13
	0.5 hr before	8.74 ± 2.48	21.59 ± 2.85	13.00 ± 2.79	0.58 ± 0.12	-0.03 ± 0.03	11.08 ± 2.51
	+stim	9.83 ± 2.55	24.41 ± 3.25*	15.90 ± 2.72*	1.02 ± 0.30*	0.26 ± 0.20	25.61 ± 8.13*
	0.5 hr after	10.35 ± 2.38	24.47 ± 2.89	16.24 ± 2.28	0.60 ± 0.10	0.18 ± 0.13	18.27 ± 5.36
Acetic acid	0.5 hr before	8.64 ± 2.45	22.12 ± 3.87	10.96 ± 2.14	0.38 ± 0.05	-0.07 ± 0.02	6.31 ± 0.85
	-stim	8.73 ± 2.25	23.57 ± 3.52	12.12 ± 2.23	0.41 ± 0.07	-0.06 ± 0.01	6.95 ± 1.27
	0.5 hr after	8.97 ± 2.52	23.10 ± 3.46	11.62 ± 2.43	0.36 ± 0.07	-0.04 ± 0.01	6.26 ± 1.47
	0.5 hr before	7.74 ± 2.08	23.89 ± 1.70	12.00 ± 2.15	0.30 ± 0.04	0.03 ± 0.04	6.54 ± 0.96
	+stim	11.11 ± 3.10	26.90 ± 2.88	17.62 ± 3.26*	0.80 ± 0.23*	0.03 ± 0.05	16.67 ± 4.40*
	0.5 hr after	10.09 ± 2.75	26.62 ± 2.35	15.21 ± 2.61	0.59 ± 0.16	0.05 ± 0.09	12.69 ± 4.83

BP: basal pressure, MP: maximum pressure, TP: threshold pressure, VV: void volume, RV: residual volume, ICI: intercontraction interval, -stim: without stimulation, +stim: spinal nerve stimulation. *P < 0.05, paired Student t-test for intragroup comparison to control “before stimulation”.

**Figure 2:**
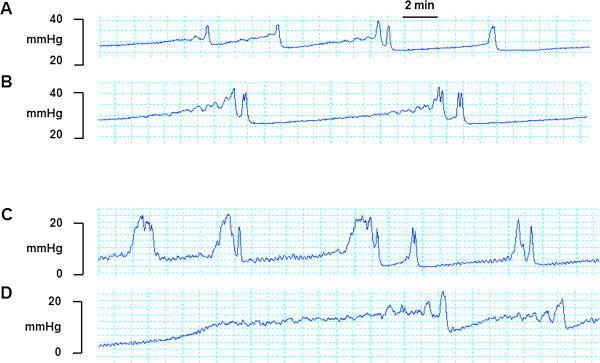
Raw traces of representative cystometric pressure recording (mmHg) before (A and C) and during threshold intensity of spinal nerve stimulation (B and D) at 10 Hz, pulse width 0.1 ms in saline- (A and B) or acetic acid- (C and D) infused rats.

Bilateral SN stimulation increased the bladder capacity, as indicated by significant increases in TP (Figure 2B and 2D; Table 2), VV (Table 2) and ICI (Figure 2B and 2D) when the urinary bladder was infused with saline or 0.3% acetic acid (irritated bladder). There are no signs of retention associated with neuromodulation judged by the lack of increases in estimated RV, and BP before, during and after the nerve stimulation in saline and acetic acid-treated rats. There was also no decreased MP associated with neuromodulation. In contrast, the MP slightly increased during stimulation in saline treated rats.

Maximal inhibitions were caused by SN stimulation (increases in VP and ICI) during the stimulation, and the effects did not persist after termination of the stimulation. SN stimulation increased the VV and ICI from 0.41 ± 0.05 ml to 1.02 ± 0.30 ml (217%), and from 7.64 ± 1.01 min to 25.61 ± 8.13 min (265%), respectively. The percentage increases of VV (383%) and ICI (350%) in the acetic acid-treated group were not significantly higher than those in saline-treated group (P > 0.05, Student t-test for intergroup comparison).

## Discussion

Acetic acid induces bladder hyperactivity with decreases in TP, VV and ICI. These excitatory effects are significantly inhibited by bilateral SN stimulation at 10 Hz. Preclinical studies on the effect of neuromodulation on bladder activity have used a wide range of stimulation parameters (frequency, intensity, duration) and different stimulation sites. In the current study, an attempt was made to use optimal stimulation parameters and optimal stimulation site. Bilateral stimulation, which is more effective than unilateral stimulation (8) was also used. Substantial changes in several parameters of urodynamic function during stimulation have been observed. We also show that SN stimulation for one hour increases functional urinary bladder capacity in both saline and acetic acid infused rats. The ability of neuromodulation to influence bladder function does not depend on the presence of bladder irritation, although there is a trend to a greater effect in the acetic-acid infused rats. Therefore, the action of neuromodulation appears to be mediated via interference with the normal micturition pathway.

It has been reported that infusion of acetic acid into the bladder induces irritation of the urothelium, stimulates nociceptive afferent fibers, and induces an inflammatory reaction. This results in a reduction in bladder capacity, and a consequent increase in contraction frequency, and other indices of bladder hyperactivity [9]. The present study demonstrated that intravesical infusion of acetic acid produced an excitatory effect on bladder micturition reflex with decreases in TP, VV and ICI.

Acetic acid produced an excitatory effect which stabilized at 2 hrs after the beginning infusion and lasted for at least 4 hrs. At the onset of an acute inflammation there are important changes in the afferent innervation of the urinary bladder [10-13]. In the inflamed bladder, even small distensions produce greater than normal rises in intravesical pressure, so that the micturition threshold is reduced. These changes develop shortly after contact of the chemical irritants with the bladder urothelium; the latency of onset correlates closely with the altered afferent nerve activity. Animals with experimentally induced cystitis show several sensory and reflex changes that are similar to those seen in humans presenting with cystitis [9]. In the current study, the hyperactivity induced by intravesical administration of acetic acid was used to mimic, in the rat, the human condition of detrusor overactitvity. This excitatory effect was significantly attenuated by spinal nerve stimulation.

By comparing Figure 2B and 2D with Figure 2A and 2C there appears to be a delayed onset of the micturition cycle activation. The increase from basal bladder pressure to the threshold pressure required to induce micturition was increased by SN stimulation in either saline or acetic acid treated rats. It seems possible that SN stimulation attenuates bladder afferent signals or interferes somehow with the first phase of the micturition cycle. There was no overflow incontinence during this prolonged interval of elevated bladder pressure before micturition occurred. In contrast, SN reversed the incontinence (urine dripping or leaking) caused by intravesical acetic acid infusion in two rats (data not shown).

Spinal nerve stimulation failed to increase basal bladder pressure or decrease the maximum pressure developed during micturition. This suggests that the SN mediated neuromodulation does not directly depress the contractility of detrusor smooth muscle at the motor threshold intensity.

Several other rat models have been utilized to evaluate the effects of neuromodulation of experimentally induced detrusor overactivity via electrical stimulation of the SN [14]. For instance, neuromodulation reduced the frequency of micturition in rat models of cystitis induced by intravesical administration of either hydrochloric acid to neuromodulation for 3 weeks, [15], turpentine oil isovolumetric model to stimulation for several mins, [16], or 48 hrs post i.p. injection of cyclophosphamide to stimulation at 20 Hz for 45 min, [2]. In spinally transected rats, sacral root stimulation abolished bladder hyper-reflexia and attenuated the rise in neuropeptide content of the L6 dorsal root ganglion [17] and expression of c-fos gene [18] and vanilloid receptor 1 [19]. Chronic sacral nerve stimulation significantly eliminated non-voiding contractions in a rat model of bladder outlet obstruction without changing bladder capacity [20]. All these studies demonstrated the ability of SN stimulation to inhibit elevated bladder activity, although it is not known whether optimal electrical stimulation parameters were used for the particular experimental conditions in each study and whether stimulation was applied at the optimal level of the spinal cord. Most studies evaluated the improvement following a chronic period of stimulation.

Acute effects on urodynamic functions during neuromodulation have been reported. Giuliano et al. [2] reported an increase in TP in saline treated rats and decreases in frequencies of either voiding contraction or non-voiding contractions in cyclophosphamide treated rats to SN stimulation on left L6 and right S1 at 20 Hz. Based on our BRC studies, stimulation at 20 Hz would not be expected to produce maximal bladder effects [3,4]. In the current study, we observed larger and additional changes, increases in TP, VV, and ICI in both saline treated rats and acetic acid treated rats during a 1 hour electrical stimulation at the L6 SN.

Clinically sacral root neuromodulation for patients with urge incontinence results in a 15-30% increase in cystometric capacity [21]. The parameters used clinically may not be optimized. We would assume that clinical benefit would improve if optimal parameters are evaluated and applied clinically in patients.

Based on clinical observations in humans, neuromodulation leads to lasting continence, e.g. the patients remain continent even when stimulation is off. However, in the current study, the effect to increase bladder capacity seems not to continue once stimulation is terminated. This may explain the different observations from other rat studies using chronic sacral nerve stimulation where SN stimulation eliminates non-voiding contractions more significantly than alters the urodynamic parameters [20]. Whether the altered urodynamic function in response to acute 1-hr stimulation leads to a sustained bladder inhibition with a different profile than long term stimulation or shares a similar mechanism seen in OAB patients under sacral neuromodulation therapy should be addressed in future studies.

We previously reported that SN stimulation inhibits the BRC frequency and often temporarily eliminated the rhythmic voiding contractions [3,4]. We argued that this “shutdown” of bladder contractions results from an action on the bladder afferent pathway since a similar effect is produced by different agents, acting by several different molecular mechanisms [22-24]. ‘Shutdown’ of the BRC is a consequence of an increased pressure threshold for initiation of contractions since further saline can re-establish the BRC even in the presence of electrical stimulation [3]. Our current results support this hypothesis, since spinal nerve stimulation increased functional bladder capacity, as measured by increased TP, VV and ICI in the cystometry model.

The effect of SN stimulation may be slightly greater in bladder irritated as compared to normal rats but the difference is not statistically significant in our test. A comparison of the clinical efficacy of neuromodulation in overactive versus normal bladder is not available, since sacral neuromodulation has not been tested in human subjects with normal bladder function. The hypothesized mechanisms of action of neuromodulation appear to involve an alteration of the transmission of sensory input from the bladder to the central nervous system, possibly activating fast conducting fibers [25,26], and to modulate neuronal circuitry in both normal and overactive bladder states.

Cystometry has been carried out in either anesthetized or conscious rats. Under conscious conditions, micturition controls are influenced by conscious brain processes. The activity of the hypogastric nerve in conscious rats appears to be stimulated by restraint [9]. Differently in anesthetized rats the neuronal circuitry involved in reflex micturition is mediated by involuntary neural mechanisms [27]. There are more facilitatory actions of capsaicin-sensitive afferent input on micturition in anesthetized rats than awake rats [28]. Therefore, anesthetized cystometry serves as a good tool to study bladder hyperactivity. However urethane influences the bladder dynamics since it alters synaptic transmission [9]. The study was performed in urethane anesthetized rats. Further experiments using conscious cystometry in chronic models of bladder overactivity will target the mechanisms by which neuromodulation acts to relieve the symptoms of overactive bladder.

## Conclusions

Neuromodulation counteracts the bladder hyperactivity and noxious input induced by chemical cystitis. Although it suppresses bladder activity in normal rats, our current results show neuromodulation to be effective in attenuating a hyperactive as well as normal bladder.

## Abbreviations

SN: Spinal nerve; BRC: Bladder rhythmic contraction; Tmot: Motor threshold; BP: Basal bladder pressure; MP: Maximum pressure; TP: Threshold pressure; VV: Void volume; RV: Residual volume; ICI: Inter-contraction interval.

## Competing interests

All authors are employees of Medtronic Inc. The research was supported by Medtronic Preclinical Research Funding.

## Authors’ contributions

Conception and design: XS. Acquisition of data: AN. Analysis and interpretation of data: XS, AN. Drafting the manuscript: XS. Revising it critically for important intellectual content: XS, DEN. Final approval of the version to be published: XS, AN, DEN. All authors read and approved the final manuscript.

## Pre-publication history

The pre-publication history for this paper can be accessed here:

http://www.biomedcentral.com/1471-2490/13/70/prepub
